# Carcinoid tumor arising from a mature cystic teratoma of the ovary: A case report

**DOI:** 10.1097/MD.0000000000041557

**Published:** 2025-02-14

**Authors:** Weitao Huang, Xiaowei Han, Jinhong Zhou, Guozheng Zhang

**Affiliations:** a Department of Radiology, The Quzhou Affiliated Hospital of Wenzhou Medical University, Quzhou People’s Hospital, Quzhou, China; b Department of Ultrasound, The Quzhou Affiliated Hospital of Wenzhou Medical University, Quzhou People’s Hospital, Quzhou, China.

**Keywords:** carcinoid tumor, case report, mature cystic teratoma

## Abstract

**Rationale:**

Carcinoid tumors arising from mature cystic teratomas of the ovary (MCTO) are exceptionally rare and often misdiagnosed as benign teratomas on imaging studies, which can lead to delayed or inappropriate treatment. The purpose of this study is to raise awareness of this rare condition by presenting a case of a carcinoid tumor originating from MCTO, highlighting its unique imaging features and clinical presentation, and discussing the importance of accurate preoperative diagnosis.

**Patient concerns:**

A 59-year-old woman was admitted to the hospital following an ultrasound physical examination that revealed an anterior uterine parenchymal mass. She exhibited no obvious abnormal clinical symptoms.

**Diagnosis:**

CT and MRI of the pelvis revealed a cystic-solid soft tissue mass with small calcified foci and a significant cystic fatty component. There was a marked enhancement of the solid component in the pelvis off to the right, leading to the consideration of a teratoma.

**Interventions:**

The final clinical preoperative diagnosis was an ovarian teratoma. Pelvic tumor resection was performed after the exclusion of contraindications to surgery. Postoperative pathology revealed a carcinoid tumor arising from MCTO.

**Limitations:**

This study is limited by the fact that it reports a single case, which makes it difficult to generalize the findings to a larger population. Additionally, the rarity of this condition means that there is limited literature available for comparison, which could provide a broader understanding of the potential challenges in diagnosing similar cases. The retrospective nature of the case also restricts the ability to conduct a more extensive analysis of potential risk factors.

**Outcomes:**

The patient did not receive adjuvant therapy post-surgery. During the 36-month follow-up period, the tumor showed no signs of recurrence or metastasis. The follow-up was concluded without further complications, indicating a favorable outcome.

**Lessons:**

Mature cystic teratoma is a common ovarian tumor, but its association with carcinoid tumors is very rare and prone to preoperative misdiagnosis. The imaging presentation of this case is characteristic and has significant diagnostic implications for this disease.

## 1. Introduction

Mature cystic teratoma of the ovary (MCTO) is a common benign ovarian tumor. Malignant transformation of any of its components, occurring in 0.06% to 2% of cases,^[1]^ is relatively rare. MCTO is composed of mature tissues derived from the ectoderm, mesoderm, and endoderm, and any of these tissue components can undergo malignant transformation. Consequently, the pathological types of MCTO are complex, with squamous carcinoma being the most common, followed by adenocarcinoma. Other less common malignant transformations include sarcoma, basal cell carcinoma, carcinoid tumor, and melanoma.^[2,3]^ Carcinoid tumors, in particular, are sporadic, accounting for <0.1% of ovarian malignancies.^[4]^ The mechanism of the malignant transformation of carcinoid tumors associated with ovarian teratomas is still unclear.^[5]^

The typical clinical symptoms of MCTO mainly include abdominal pain and distension, often accompanied by elevated levels of tumor markers on preoperative examination.^[6]^ Of note, patients with carcinoid lesions usually exhibit no obvious clinical symptoms or signs and are often discovered due to the presence of a pelvic mass. Some patients may experience carcinoid syndrome, which includes skin flushing, watery diarrhea, bronchospasm, capillary dilation, and cardiac damage syndrome. These symptoms are related to the secretion of 5-HT and kinins by carcinoid tumors.^[7]^ Herein, our patient presented with a pelvic mass and no other symptoms or signs. Ovarian cystic mature teratoma with carcinoid tumor is frequently misdiagnosed as a simple teratoma, requiring postoperative pathology for a definitive diagnosis.

## 2. Case presentation

**Figure 1.**
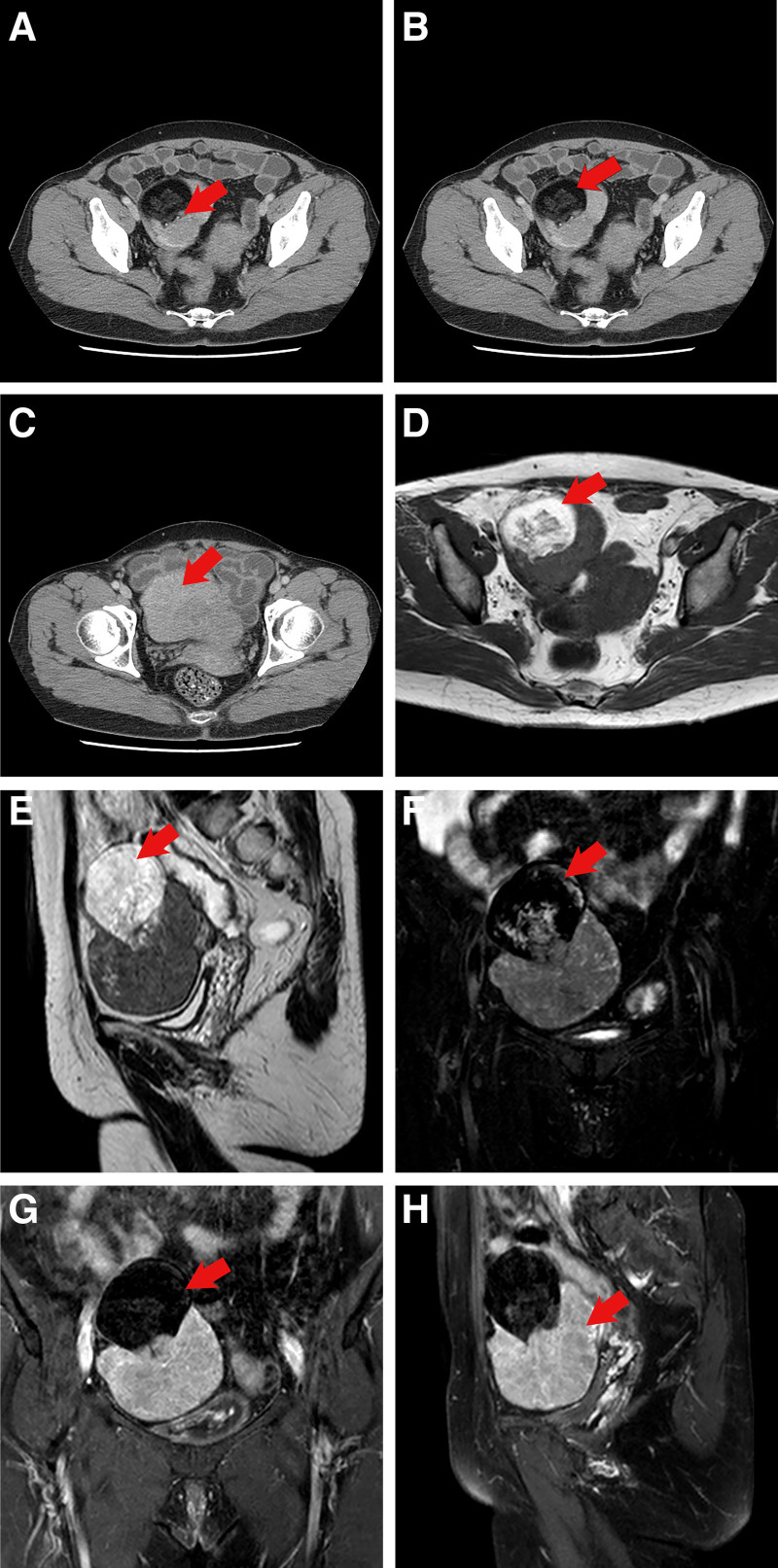
(A–H) CT and MRI imaging of the tumor. (A) A cystic-solid mixed-density shadow was seen on the right side of the pelvis, with a small amount of calcification (arrow). (B) The cystic component exhibited fat density (arrow). (C) The solid part of the lesion demonstrated marked and more homogeneous enhancement on scanning (arrow). (D) The cystic part of the T1WI sequence showed a high-signal area (arrow). (E) The cystic part of the T2WI showed a high-signal shadow (arrow). (F) The signal of the cystic part was significantly attenuated on compressed fat scanning (arrow). (G) Significant homogeneous enhancement of the solid portion following enhancement (arrow), while the cystic portion was not enhanced. (H) Significant homogeneous enhancement of the solid portion following enhancement (arrow), while the cystic portion was not enhanced.

**Figure 2.**
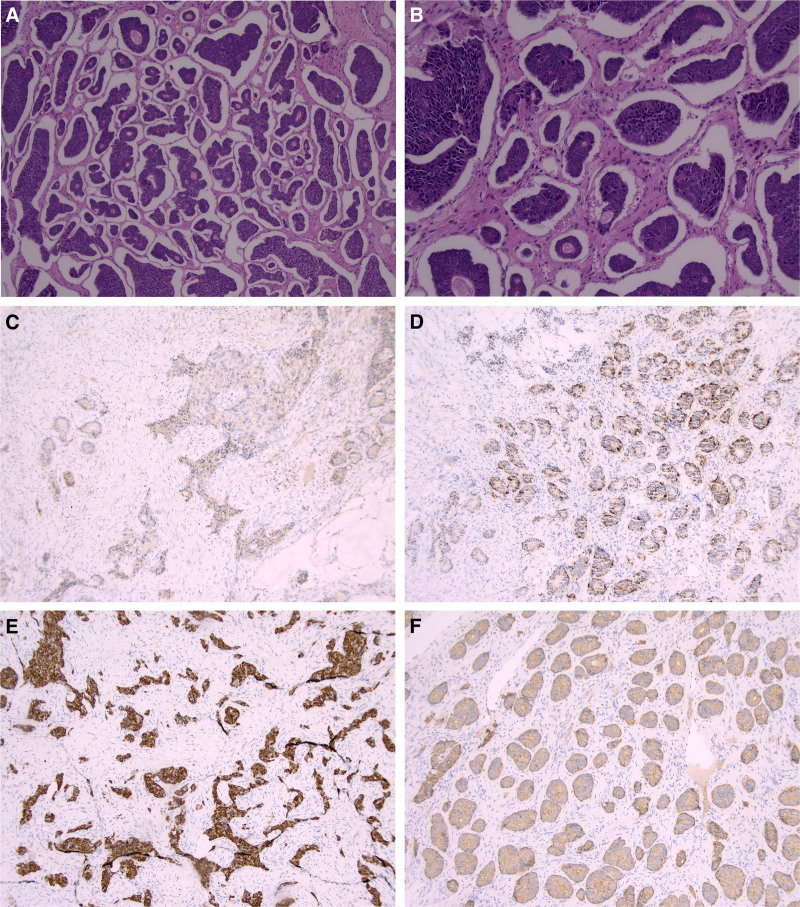
(A–F) Pathological findings and immunohistochemical staining results. (A) H&E, ×100, (B) H&E, ×200, (C) CD56, (D) CgA, (E) CK, and (F) Syn (IHC, ×200).

The patient, a 59-year-old woman, and a routine ultrasound (US) examination accidentally revealed a solid mass in the anterior wall of the uterus. She entered menopause 10 years ago and had no postmenopausal vaginal bleeding or discharge during that time. During the course of the disease, she experienced no urinary frequency, urgency, difficulty in urination, abdominal distension, diarrhea, constipation, skin flushing, capillary dilation, bronchospasm, or other symptoms. Moreover, she did not have nausea or emaciation. Physical examination revealed a 10 cm diameter mass in the right adnexa, which was hard with poor mobility, and the left adnexa was indistinguishable. Laboratory examinations showed no significant abnormalities. Abdominal CT revealed a cystic-solid mixed-density shadow in the right pelvis (Fig. 1A), measuring approximately 6.7 × 7.7 × 9.8 cm, with clear boundaries, small calcified shadows, and fat density in the cystic component (Fig. 1B). The enhancement scan showed a homogeneous strengthening of the solid part of the lesion (Fig. 1C), and the diagnosis was considered to be a teratoma. Pelvic MRI revealed a cystic-solid mass in the right adnexal region, with the cystic part showing a high signal in T1WI and T2WI (Fig. 1D and E) and a low signal after fat compression scanning (Fig. 1F). The size was approximately 6.7 × 7.7 × 9.8cm, and the solid part of the enhancement scan showed apparent strengthening with a smooth edge (Fig. 1G and H). The final preoperative diagnosis was ovarian teratoma. After excluding surgical contraindications, a total abdominal hysterectomy with bilateral oophorectomy and salpingectomy was performed. Intraoperatively, the right ovary was enlarged and whitish, with no obvious papillae and a cystic-solid appearance. Postoperative pathology revealed a mature cystic teratoma with a carcinoid tumor of the ovary (Fig. 2A and B). Immunohistochemistry results showed weak positivity for ER and PR, negativity for WT-1, CA125, and P53, small foci positivity for CEA, CK7, CK20, and CDx-2, 1% positivity for Ki-67, positivity for CK, Vim, CD99, and CD56, individual positivity for a-Inhibin and Calretinin, partial positivity for EMA, and positivity for CgA and Syn (Fig. 2C–F). No postoperative adjuvant therapy was administered, and the tumor did not recur or metastasize during the 36 months of follow-up.

## 3. Discussion

Malignant transformation in mature cystic teratomas of the ovary is a rare occurrence, with carcinoid tumors being exceptionally uncommon and usually documented through case reports. These carcinoid tumors in ovarian teratomas are believed to originate from neuroendocrine cells found in the epithelium of the gastrointestinal or respiratory tract.^[8]^ Carcinoid tumors often secrete various substances, including serotonin, histamine, catecholamines, prostaglandins, and vasoactive peptides, which can lead to carcinoid syndrome. In contrast, our patient did not develop carcinoid syndrome, making the initial diagnosis challenging.

There are no clear diagnostic criteria for cystic mature teratoma with carcinoid tumor of the ovary, but certain factors should be considered according to previous reports. Firstly, the age of onset for primary carcinoid tumors of the ovary varies widely, typically occurring in menopausal or postmenopausal women,^[9]^ as was the case with our middle-aged patient. Secondly, tumor size is an important indicator: tumors larger than 9.9 cm have an 86% sensitivity for malignancy, and retrospective studies have shown that 78.7% of malignant MCTO tumors exceed 10 cm in diameter.^[10,11]^ The tumor in the present case had a maximum diameter of approximately 11 cm. Notably, imaging findings provide valuable clues for diagnosis. Ovarian cystic mature teratomas usually appear as cystic or cystic-solid masses with relatively uniform cyst wall thickness, possible calcification, lipid density, and well-defined borders. Meanwhile, malignant cases often show unevenly thickened cyst walls exceeding 0.3 cm, irregular outer edges, and blurred borders, frequently accompanied by hemorrhage and necrosis. In addition, solid or cystic-solid protrusions within the cyst wall, known as head nodules, are typically 1.0 to 4.5 cm in diameter, with malignant nodules being often larger, exceeding 5.0 cm.^[9]^ The enhancement pattern is also very indicative. While cystic mature teratomas may contain some solid components, they usually show no or mild enhancement on scans. In contrast, malignant cases exhibit irregular, significant enhancement or thickened and twisted blood vessel shadows. Malignant cystic mature teratomas of the ovary can directly invade adjacent tissues, as indicated by the disappearance of the fat layer between the tumor boundary and surrounding organs and occasionally enlarged lymph nodes. In our case, in addition to typical teratoma features, imaging revealed a 7.5 cm solid nodule with necrosis. The solid component showed uneven enhancement after contrast administration, with thickened and twisted blood vessels observed at the periphery, in line with literature reports.

In conclusion, carcinoid tumors arising from MCTO can occur in patients of any age, often presenting with atypical clinical symptoms that may or may not include carcinoid syndrome. Imaging typically shows features of a teratoma, such as a tumor diameter >10 cm, nodules larger than 5.0 cm, or a non-uniformly thickened cystic wall exceeding 0.3 cm. There is a significant enhancement of the solid component after contrast administration and the presence of thick, twisted blood vessels, with or without invasion of surrounding tissues and organs. Therefore, a definitive diagnosis relies on postoperative histological examination and immunohistochemical analysis.

## Author contributions

**Conceptualization:** Weitao Huang.

**Data curation:** Weitao Huang, Jinhong Zhou.

**Formal analysis:** Weitao Huang, Guozheng Zhang.

**Funding acquisition:** Xiaowei Han, Jinhong Zhou, Guozheng Zhang.

**Project administration:** Weitao Huang.

**Software:** Weitao Huang.

**Validation:** Weitao Huang.

**Visualization:** Guozheng Zhang.

**Writing – original draft:** Weitao Huang.

**Writing – review & editing:** Guozheng Zhang.
